# Dynamic tuning of optical absorbance and structural color of VO_2_-based metasurface

**DOI:** 10.1515/nanoph-2023-0169

**Published:** 2023-06-12

**Authors:** Tao Cheng, Yukuan Ma, Huanhuan Zhao, Tianhao Fei, Linhua Liu, Jia-Yue Yang

**Affiliations:** Optics & Thermal Radiation Research Center, Institute of Frontier and Interdisciplinary Science, Shandong University, Qingdao, 266237, P.R. China; College of Electronic Information, Sichuan University, Chengdu, 610000, P.R. China; School of Energy and Power Engineering, Shandong University, Jinan, 250061, P.R. China

**Keywords:** dynamic tuning, Fabry–Perot resonance, metasurface, phase change materials, structural color, transition metal dichalcogenides

## Abstract

Vanadium dioxide (VO_2_) is an attractive thermal-control material exhibiting low thermal hysteresis and excellent temperature cycling performance. However, the deficiencies including weak spectral shift and narrow-band absorption during insulating-metallic transitions hinder its application in optoelectronics. The transition metal dichalcogenides (TMDs) can provide a promising solution with their high dielectric properties and robust optical coupling. Here, we report a MoS_2_/VO_2_/Au/Si metasurface and investigate the dynamic tunability of its optical absorbance and structural color upon heating via spectroscopic ellipsometry measurements and numerical simulations. The first-principles calculations reveal that the dielectric absorptions of metallic and insulating VO_2_ oppositely response to temperature, closely related to the difference in the transitions of O-2p states. Finite-element simulations reveal that the introduction of MoS_2_ nanostructure induces more absorption peaks by 2∼3 and achieves strong absorption in the full wavelength range of visible light. The Fabry–Perot (F–P) resonance is the critical factor for the optimized optical absorption. The structural color is sensitive to environmental perturbations at high-*ε* state of VO_2_, lower oblique incidence angles, and heights of MoS_2_. This work seeks to facilitate the spectral modulation of phase change metamaterials and can be extended to photoelectric detection and temperature sensing applications.

## 1 Introduction

Optical metasurfaces, classified as artificial nanostructured compounds, can exhibit unconventional responses to light in contrast with natural materials, such as high efficiency [1, 2], high bandwidth [3–5], high-quality factor Q [6–8], and perfect absorption [9–11]. Integration of phase change materials (PCMs) with typical optical metasurfaces [12–14] can achieve the real-time tuning of optical resonances and offer promising prospects for multifunctional optoelectronic devices [15, 16]. Vanadium dioxide (VO_2_) is an excellent thermal-control material attributed to its low phase-change temperature (around 68 °C), low thermal hysteresis, and high performance in switching cycles. Boyce et al. [13] investigated the plasma dynamic tuning of Au/sub-10 nm-thick VO_2_ film structure and confirmed the remarkable stability of the spectra at more than 10 temperature cycles. Kepic et al. [17] constructed VO_2_ nanoantennas with tunable Mie resonance in the visible range, exhibiting significant scattering and extinction modulation depth. Ke et al. [18] utilized VO_2_ nanoparticles to achieve distinct temperature-response switching. However, VO_2_ faces several obstacles, such as weak spectral shift under two states, narrow absorption range, low Q-factor, limited solar modulation [19], and unfavorable brown color. It thus hinders its applications in photoelectric detection [20], perfect absorption, and structural color.

Though high-*ε* materials have been introduced to enhance the absorption [5], yet studies on the absorption features’ tunability still lack. The transition metal dichalcogenides (TMDs) provide one promising solution because of their excitonic absorption, high photoresponsivity, and robust optical coupling [21–25]. It exhibits potential for optical absorption [26], photoluminescence [27, 28], and detectors [29]. Previous works [20, 30, 31] have successfully achieved resonant optical absorption by coupling TMDs with VO_2_ multilayer metamaterials. Yan et al. [20] achieved active tuning of the collective Mie resonances from silicon nanoparticle clusters by constructing a new platform of VO_2_ thin film with a tungsten disulfide (WS_2_) sheet. Hou et al. [30] combined molybdenum disulfide (MoS_2_) with VO_2_ to modulate its photoluminescence. Chen et al. [31] developed a novel model of tunable broadband near-infrared absorber with the help of MoS_2_ coupled with VO_2_. Most studies have focused on the performance changes by additionally adding TMDs to the VO_2_ composite structure. However, the specific ability of TMDs to improve the spectral absorption of VO_2_ and the underlying physical mechanisms have been rarely reported, which underestimates the development potential of TMDs–VO_2_ composite devices. Furthermore, improving the spectral tunability of the VO_2_ composite structure in the visible range can enhance the dynamic control of the structural color [32, 33]. The structural color originates from the interaction between light and complex micro- and nanostructures, involving fundamental optical processes such as diffraction, interference, and scattering [34], and has excellent color and gloss stability and good thermal and chemical resistance. Zhao et al. [32] improved the dynamic tuning of the structural color of VO_2_ by varying parameters such as the layer thickness of metal and VO_2_. Kim et al. [33] improved the reflected color purity of VO_2_ tuning by constructing nanoparticle absorbers. Yet, specific studies of TMDs to modulate VO_2_ composite structures’ color remain untapped.

Herein, we have investigated the spectral tuning possibility and physical mechanisms of MoS_2_/VO_2_/Au/Si metasurface in the visible range with the help of spectroscopic ellipsometry (SE) experiments, density functional theory (DFT), and finite-element method (FEM) simulations. First, a multilayer VO_2_/Si/Au structure is fabricated, and the spectral properties are obtained with SE. Then the contribution of O-2p electrons in VO_2_ to dielectric function at different phases is shown using DFT. The dependence of absorption on temperature and VO_2_ thickness is investigated with FEM, obtaining the excitation conditions of Fabry–Perot (F–P) resonance. Furthermore, the MoS_2_/VO_2_/Si/Au metasurface is designed and found to have a broader range of solid absorption and richer spectral features with modulating incident angle and MoS_2_ morphology than the multilayer structure. The electromagnetic field results reveal that the F–P resonances in the VO_2_ and MoS_2_ layers play a critical role in spectral regulation. Finally, the structural color is studied as an extended application based on spectral modulation and exhibits different sensitivity to temperature, oblique incidence angles, and heights of MoS_2_. This work aims to facilitate tuning the VO_2_ metamaterial active coupling light and dynamic structural color.

## 2 Experimental & computational methodology

### 2.1 Structural design

**Figure 1:**
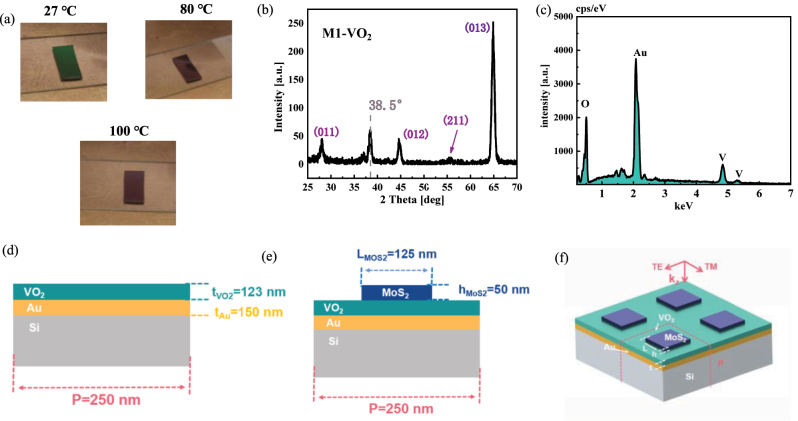
Characterization of the prepared samples and model parameters for calculation. (a) The real optical photograph images of the fabricated VO_2_/Au/Si multilayer structure at 27 °C, 80 °C, and 100 °C, respectively. (b) XRD results of VO_2_/Au/Si multilayer structure at grazing incidence. (c) EDS spectrum of VO_2_/Au/Si multilayer structure. (d) Optical schematic of the hierarchical structure. (e) Parametric diagram and (f) global schematic diagram of MoS_2_/VO_2_/Au/Si metasurface.

The VO_2_/Au/Si hierarchical structure was fabricated by the magnetron sputtering method as shown in Figure 1a. Real photographic images of the hierarchical structure before and after the phase transition are presented. The structure appears lime green at room temperature and yellowish brown after the phase transition. The sample was characterized by XRD (Rigaku SmartLab 3 kW) with Cu K1 irradiation at a scanning rate of 3°/min with 2*θ* ranging from 25° to 70°. In the test, the grazing incidence was chosen at an incidence angle of 0.5° because that the high energy of normal incident light easily penetrates the film and reaches the substrate, thus causing a large disturbance to the final result. The peaks at 28.03°, 44.5°, 55.5°, and 64.8° in Figure 1b correspond to the (011) [35], (012) [36], (211) [37], and (013) [36] planes of VO_2_, respectively, confirming the existence of monoclinic (M_1_) phase transition of VO_2_. It is worth noting that there is a 38.5° peak, which is not strictly an Au diffraction peak ((111), 37.9° [35]) nor a VO_2_ diffraction peak ((020), 39.9° [37]). This is a mixed peak of the two elements caused by the energy breakdown through the VO_2_ film to the Au surface due to the excessive intensity of the light source and the thin thickness of the VO_2_ film. The diffraction peaks of other impurities or phases were not detected except for the diffraction peaks of M_1_-VO_2_ films. The surface morphology (Figure S1) and energy dispersion (Figure 1c) of the samples were also investigated by scanning electron microscope (SEM) – energy dispersive spectroscopy (EDS) model SU8220. The results confirm the presence of V and O elements in the sample. Figure 1c also shows the Au peak caused by the energy breakdown through the VO_2_ film.

Figure 1d depicts the experimentally prepared schematic diagram of multilayer VO_2_/Au/Si. The total thickness is 1 mm, and the thickness of Au and VO_2_ is 150 nm and 123 nm, respectively. In the FEM simulation, the periodic tetragonal structure with a period *P* of 250 nm × 250 nm is used. The total thickness is set to 700 nm, ensuring that the electromagnetic waves do not penetrate the structure, so the absorption is *A* = 1−*R*. Figure 1e and f shows the MoS_2_/VO_2_/Au/Si metasurface, where the MoS_2_ shape is square with default side length and height of *L* = 125 nm and *h* = 50 nm, respectively. The main polarization directions of the light source are transverse electric (TE) and transverse magnetic (TM) directions.

### 2.2 Experiments and simulations

The reflectivity and temperature-dependent dielectric function of the VO_2_/Au/Si hierarchical structure was measured with the RC2 ellipsometer purchased from J.A. Woollam Company. In the high-temperature test, the Linkam heat cell heated the system. The measured angle of incidence was set to 70°. Nitrogen gas was continuously introduced during heating to remove oxygen from the heating chamber to prevent oxidation. The ellipsometric parameters of *ψ* and Δ were first obtained by RC2 ellipsometer. Each layer was modeled separately for the hierarchical structure to extract its dielectric function and thickness. The silicon substrate layer was modeled first, followed by an intermediate layer of thin-film gold with a thickness of 150 nm. Finally, the VO_2_ layer was modeled with the optical model B-spline [38] to improve the fitting accuracy of this layer, and the thickness fitting option was turned on. The dielectric functions of Si, Au, and VO_2_ at temperatures ranging from 30 °C to 300 °C in the spectral range of 300 nm–1600 nm were obtained and the thickness of VO_2_ obtained by fitting was 123 nm. A more detailed ellipsometric working principle can be found in our previous study [39].

With the SE-measured dielectric function as input, FEM simulations on radiative properties of multilayer structures were performed. Floquet periodic boundary conditions were used in the *X* and *Y* directions to mimic the composite structures, and perfect matching layers (PML) boundary conditions were used in the *Z* direction to truncate the simulation volume. The thickness of PML was set to 1/4 wavelength and the convergence mesh to 1/5 wavelength, respectively.

The impedance transformation method can be applied to analyze the absorption properties of the structure, and the impedance *Z* of the absorber and impedance *Z*_0_ of free space are calculated following [40, 41]

(1) $$Z=\pm \sqrt{\frac{{\left(1+S_{11}\right)}^{2}-S_{21}^{2}}{{\left(1-S_{11}\right)}^{2}-S_{21}^{2}}}$$

(2) $$Z_{0}\left(\lambda \right)=\sqrt{\frac{\mu \left(\lambda \right)}{\varepsilon \left(\lambda \right)}}$$

where *S*_11_ and *S*_21_ are the reflection and transmission coefficients, *μ*(*λ*) and *ε*(*λ*) are the magnetic permeability and dielectric function of the structure at the wavelength, respectively. The closer the impedance of the absorber is to *Z*_0_ = 1, the stronger its absorption capacity.

## 3 Results and discussions

### 3.1 VO_2_/Au/Si hierarchical structure

**Figure 2:**
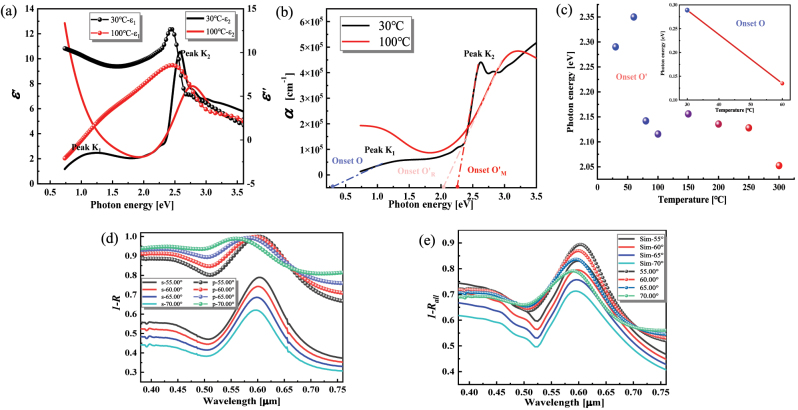
Ellipsometric measurements: (a) VO_2_ dielectric function under two phases, (b) VO_2_ absorption coefficients under two phases and the location of the marked of absorption onset, (c) absorption onset at different temperatures, (d) absorptance under *s* (TE) and *p* (TM) polarization, and (e) the comparison between experimental measurements and FEM calculations.

We first investigate the dielectric function of VO_2_ films in the VO_2_/Au/Si hierarchical structure. According to previous studies [42, 43], VO_2_ undergoes tetragonal rutile (R) phase to M_1_ phase transition as the undimerized vanadium atoms undergo classical Mott transition, and the crystal symmetry is broken (Figure S2). This process inherently changes the dielectric function of VO_2_, which is closely related to the atomistic and electronic structure, and Figure 2a and Figure S3 illustrate the differences. The onset of the absorption peaks of the absorption coefficient spectrum (*α*) reflects the optical band gap and can be obtained from the intersection of the tangent line of the absorption edge of the spectral absorption peak with the energy axis (*x*-axis) [44], as shown in Figure 2b. In VO_2_, the absorption onset O of main peak K_1_ (Figure 2b) is related to the indirect transition process involving O-2p and V-3d states near the Fermi energy level. And the onset O′ of peak K_2_ is contributed by the transition process of O-2p states to higher energy levels (see S4 for more details in Supplementary Information). The temperature dependence of onsets for different absorption peaks is thoroughly investigated. As seen in the inset of Figure 2c, high temperature induces a red-shift of the onset O due to Mott physics [42], i.e., the band gap energy decreases with increasing temperature. In the R phase, the onset O disappears due to the vanishing of the interband transitions between V-3d and O-2p states. On the other hand, the onset O′ of peak K_2_ blueshifts with increasing temperature in the M_1_ phase indicates an increase in the transition energy of O-2p states. However, after the phase transition occurs, there is a significant red-shift in the absorption onset O′ due to the reduced band gap and lower transition energy of O-2p states to higher energy states (Figure S4a–b). Also, there is an overall decreasing pattern of onset O′ with increasing temperature, which indicates that high temperature causes a decrease in the O-2p states transition energy in the R phase. More relevant details of DFT calculations and discussions are given in Supplementary Information S4.

**Figure 3:**
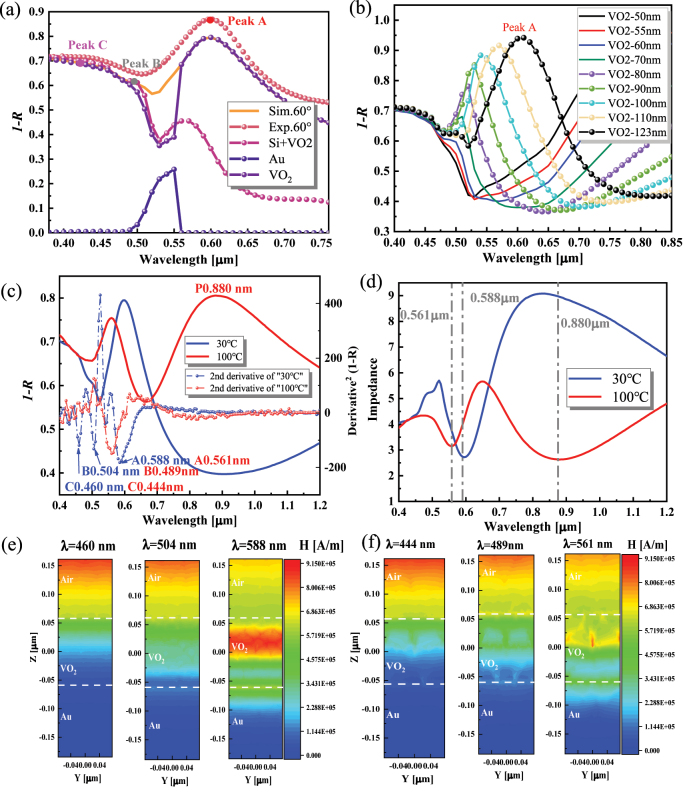
Absorption characteristics of VO_2_/Au/Si hierarchical structure. (a) The absorption at 60° incidence, including the VO_2_/Au/Si hierarchy (including simulation and experiment), the individual contribution from its components (including Au layer and VO_2_ layer), and VO_2_/Si bilayer reference structure. (b) Calculation of the thickness dependence of VO_2_ at vertical incidence. (c) Absorption of the hierarchy structure at 30 °C and 100 °C at 60° incidence. The positions of absorption peaks A, B, and C at 30 °C and 100 °C are 0.588 nm, 0.504 nm, 0.460 nm and 0.561 nm, 0.489 nm, 0.444 nm, respectively. Dotted lines plots indicate $\frac{\partial^{2}A}{\partial \lambda^{2}}$ at the corresponding temperatures. (d) Impedance ((*Z*_TE_ + *Z*_TM_)/2) of the hierarchy structure at 30 °C and 100 °C at 60° incidence. Magnetic fields of absorption peaks A, B, and C at (e) 30 °C and (f) 100 °C.

Next, the radiation characteristics of the multilayer structure are studied using FEM simulations. Since fewer metallic features are involved [45], we call the dielectric states before and after the phase transition high-*ε* and low-*ε*, respectively. Figure 2d shows a considerable polarization sensitivity in the absorption of the hierarchical structure at oblique incidence, so all the absorption mentioned in this paper is total *A*_total_ = (*A*_TE_ + *A*_TM_)/2. As seen in Figure 2e, the FEM simulation results agree well with experimental results regarding absorption peak positions, which provides favorable conditions for the peak coupling study below. However, the absolute value of the simulated absorption is lower than that of the experimental results, possibly due to the deviation of the VO_2_ thickness (confirmed by Figure 3b) chosen in the simulation from the actual experiment and errors from the material parameters.

Then, the absorption contribution of each layer in the hierarchical structure is investigated. Figure 3a shows that the VO_2_/Au/Si structure has three absorption peaks in the visible range, and they are all contributed by the VO_2_ layer, which can be confirmed by comparing the absorption of the VO_2_/Si bilayer structure. The Au layer mainly contributes to the 0.46–0.56 μm range absorption. To better distinguish the absorption features, we fit the second derivative of the absorption spectra concerning wavelength $\left(\frac{\partial^{2}A}{\partial \lambda^{2}}\right)$ referring to the previous treatment of enhanced dielectric function spectral line shapes [46, 47] (see S5 for more details in Supplementary Information). Then, the formation conditions of the F–P resonance [20] in VO_2_ and its contribution to peak A are studied. The magnetic field (Figure S6) at peak A is localized inside the VO_2_, confirming the formation of F–P resonance. Figure 3b shows that as the thickness of VO_2_ decreases, the absorption peak A blueshifts and weakens until peak A disappears at the height *h* ≈ 50 nm. It is due to the reduction of the effective cavity length and the gradual disappearance of the F–P resonance. The low thickness exhibits a narrower bandwidth, which has lower energy dissipation, as evidenced by the more muscular magnetic field strength (Figure S6).

When VO_2_ shifts from high-*ε* state to low-*ε* state, peak A blueshifts and decreases in value (Figure 3c). It is due to the weakened F–P resonance, and the magnetic field exhibits a weaker and nonuniform character, as shown in Figure 3e and f. Since *A*_TE_ is similar to *A*_total_, this paper’s magnetic and electric fields are considered under the TE wave. In the near-infrared (NIR) range, a plasmonic resonance (peak P) appears attributed to the more active surface electrons in metallic VO_2_ [31]. The solid optical interference [30] enhances the total absorption within the spectrum (Vis-NIR region), as shown in Table S1. The impedance transformation method can also be used to analyze the absorption changes of the hierarchical structure caused by the phase transition of VO_2_ [48]. As shown in Figure 3d, the low-*ε* state makes the impedance lower and closer to 1, thus increasing the absorption of the structure. The peak A related to F–P resonance of the low-*ε* state is blue-shifted concerning the high-*ε* state, while the impedance value of the low-*ε* at the dip is higher than that of the high-*ε* state, indicating a lower absorption capacity. The impedance curve is smoother in the range below 561 nm, which is shown in the absorption curve as the disappearance of the remaining peaks except peak A (Figure 3c). In addition, an impedance dip at the wavelength of 880 nm indicates the appearance of a new absorption.

### 3.2 MoS_2_/VO_2_/Au/Si metasurface

**Figure 4:**
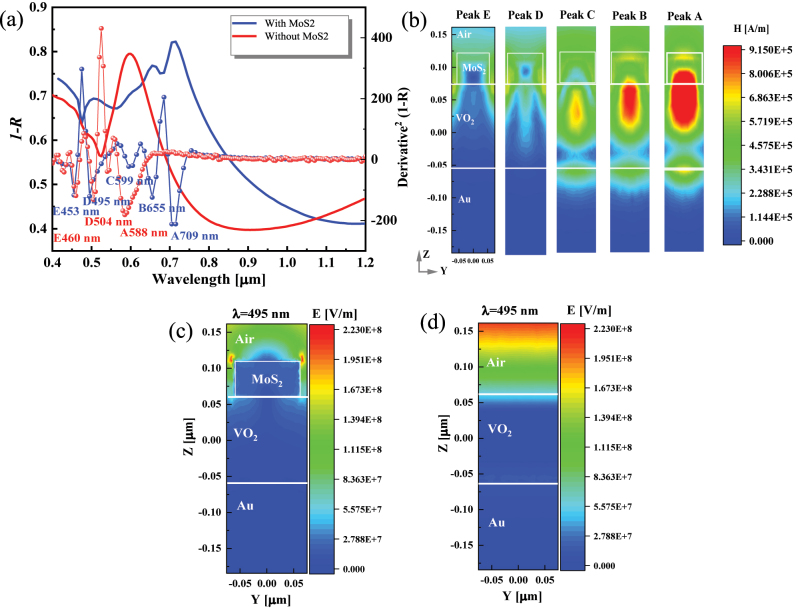
Absorption characteristics of MoS_2_/VO_2_/Au/Si metasurface. (a) Comparison of absorption of structures with and without MoS_2_ at an oblique incidence angle of 60°. The size of MoS_2_ is the default structural parameter. Dotted lines plots indicate $\frac{\partial^{2}A}{\partial \lambda^{2}}$ at the corresponding structure. (b) The magnetic fields at different peaks of the structure with MoS_2_. (c) Optical field of peak D (*λ* = 495 nm) under MoS_2_ nanostructure. (d) Optical field of D under VO_2_/Au/Si structure.

The bulk MoS_2_ has excellent excitonic polarization that can realize light–matter solid interactions [49], which helps improve the spectral tuning of the VO_2_ system. A MoS_2_/VO_2_/Au/Si simulation model is developed using the default parameter values in Figure 1c and d and studied for the radiation performance. The dielectric function of bulk MoS_2_ is available in the literature [27]. Figure 4a shows that the introduction of the bulk MoS_2_ broadens the strong absorption range and increases the absorption features, which is attributed to the high-*ε* property (see Equation (S2) in Supplementary Information) and abundant exciton absorption of MoS_2_ (Figure S3e). Peak A red-shifts, and peaks B, C, and E related to MoS_2_ excitons emerge, suggesting the enhanced average optical absorption (from 0.54 up to 0.65 in the 400 nm–1000 nm range). Peak D contributed by MoS_2_ blueshifts slightly and increases in amplitude. Figure 4b–d demonstrates the specific physical mechanism. The magnetic fields at peaks A, B, and C show a longer optical path and are located in the middle region between the lower MoS_2_ layer and the upper VO_2_ layer, demonstrating that a new F–P resonance is generated in MoS_2_ [20], where the Au layer and the top MoS_2_ act as two reflectors. The optical field at peak D is strongly localized at the MoS_2_ surface edge, and the internal field strength is also more extensive than that of the pure VO_2_ structure (Figure 4c and d), explaining the high value (see Equation (S2) in Supplementary Information) and the narrow bandwidth of peak D.

**Figure 5:**
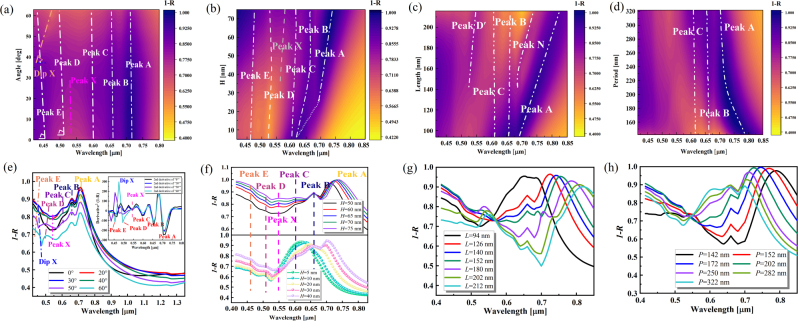
Contour map of absorption *A*__total__ dependent on structural morphology. (a) Calculated *A*_total_ contour map at different angles. (b) Calculated height-dependent absorption *A*_total_ contour map of MoS_2_ at vertical incidence. (c) Calculated length-dependent absorption *A*_total_ contour map of MoS_2_ at vertical incidence, *P* = 250 nm. (d) Calculated period-dependent absorption *A*_total_ contour map of metasurface at vertical incidence, *L* = 125 nm. (e) *A*_total_ of metasurfaces at different angles. The inset shows the $\frac{\partial^{2}A}{\partial \lambda^{2}}$ at the corresponding angles. (f) Calculated absorption at different MoS_2_ heights at vertical incidence. (g) Calculated absorption at different MoS_2_ lengths at vertical incidence, *P* = 250 nm. (h) Calculated absorption at different metasurface periods at vertical incidence, *L* = 125 nm.

Furthermore, the effect of the polarization and incidence angles on the MoS_2_ metasurface is investigated. The sensitivity of the metasurface to the polarization angle is first studied, as shown in Figure S7a–d. When averaging the absorption of the TE and TM directions at an oblique incidence angle of 60°, it is found that in the 0°–80° polarization range, the absorption shows a symmetric distribution with the polarization angle of 40° as the dividing line, i.e., the closer to 40° polarization angle, the larger the absorption of the metasurface. Then, the sensitivity of the metasurface to the incidence angle is investigated. As shown in Figure 5a and e, peak A decreases with increasing angle, while the absorption in the range from peak E to peak B position shows the first increase and then decreases with increasing angle, and the turning point of the change is near 30°. It is evidenced by the weaker optical field of peak B at a larger angle (>30°), as shown in Figure S7e. The contour plot of total absorption (contour plots of absorption in the TE and TM directions are shown in Figure S8a and S8b in Supplementary Information) shows that the dip X becomes apparent around 35°, which derives from the blueshift and decreases in the bandwidth of peaks E and D with increasing angle. The peak X (at *λ* = 544 nm) is smoothed to annihilation at an increasing angle of 50°, which the more divergent magnetic field can explain at a higher angle (Figure S7f). In a word, the incidence angles can improve the spectral modulation performance of the metasurface and realize the free transition between multiple peaks and dips.

The excitonic features of MoS_2_ determine the absorption of the metasurface, and the height affects the performance of the excitonic mode, so the optical absorption related to the height of MoS_2_ is further investigated. The finite-thickness MoS_2_ weakly increases the absorption and optical fields of the structure (Figure S9a–b). However, as the bulk MoS_2_ thickness increases to about 35 nm, peak A keeps red shifting and splitting, and its slight dip feature at *λ* = 660 nm turns into a peak (B) feature (Figure 5b and f). This is due to increased excitonic mode coupling specific gravity [50], which induces the Fano resonance. In this process, the quality of peak C (*λ* = 612 nm) also becomes apparent and no longer varies with height. The $\left(\frac{\partial^{2}A}{\partial \lambda^{2}}\right)$ shows more information on the displacement of the absorption peaks with height (Figure S9c). In addition, the absorption increases with height, and the average absorption in the visible region (380–760 nm) reaches 0.9 when *h* = 70 nm (angle = 0°). A nearly perfect absorption of 0.9992 can be achieved at *λ* = 735 nm. This is due to the increased effective cavity length and the enhancement of the optical interference [30]. The magnetic fields are more localized at the higher height in the MoS_2_ nanostructure (Figure S10), where the excitons coupling is enhanced. It proves that the F–P resonance is also generated in MoS_2_ at long wavelengths at a certain height.

The length of MoS_2_ (*L*) determines the responsiveness of the nanostructure to light. Therefore, maintaining the period *P* = 250 nm unchanged, the effect of MoS_2_ size on the metasurface’s optical absorption is investigated. Figure 5c–g show that the dominant peak A exhibits a significant red-shift and decreases in amplitude as *L* increases. When *L* increases to 140 nm, a new absorption peak N appears near *λ* = 680 nm and shows a trend of feature enhancement followed by weakening as *L* increases. This feature can be interpreted with the help of the optical field, as shown in Figure S11. At this time, the strong magnetic field is contributed by the upper surface and lower surface (MoS_2_/VO_2_ intersection) of MoS_2_. The optical field is mainly localized around the MoS_2_ bottom corner between adjacent cells, and the MoS_2_ interior also contributes a part of the field strength. In addition, when *L* increases to 142 nm, the absorption peak D′ at *λ* = 520 nm becomes gradually apparent. By observing its optical and magnetic field distribution, one can find that the peak D′ is very similar to D contributed by MoS_2_ mentioned above (see S13 for more details in Supplementary Information). Then, keeping the MoS_2_ size *L* = 125 nm unchanged, the period (*P*) variation on the metasurface absorption properties is investigated. First, the changes in the period do not produce new absorption features (peaks or dips). As the period increases, the prominent peak A blue shifts, and its broadening decreases. The peaks of exciton peaks B and C increase, and their broadening increases. By defining the filling factor FF = (*L*/*P*)^2^ [51] for the metasurface, it is observed that the absorption peak A red-shifts markedly and the exciton peaks B and C decrease with increasing filling factor.

**Figure 6:**
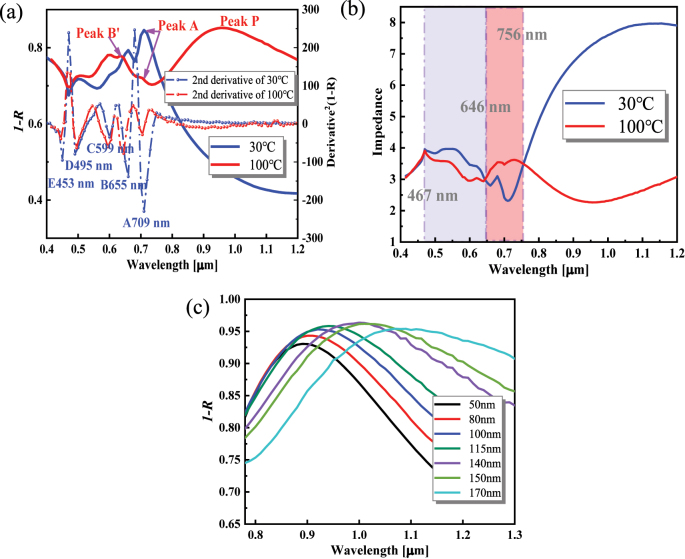
Absorption of MoS_2_/VO_2_/Au/Si metasurface effected by VO_2_ phases transition. (a) Comparison of calculated absorption in the high-*ε* state and the low-*ε* state at an oblique incidence angle of 60°. Dotted lines plots indicate $\frac{\partial^{2}A}{\partial \lambda^{2}}$ at the corresponding structure. (b) Impedance ((*Z*_TE_ + *Z*_TM_)/2) of the MoS_2_ metasurface at 30 °C and 100 °C at 60° incidence. (c) Variation of plasmonic resonance peaks with MoS_2_ side length *L*.

The effect of VO_2_ phase transition on absorption is further investigated, as shown in Figure 6a. When VO_2_ changes from high-*ε* state to low-*ε* state, the absorption peaks blueshift, and peak A couples with peak B to produce a blue-shifted peak B′ with a higher value. The quality factors Q (see Equation (S3) in Supplementary Information) [50] before and after coupling are 9.39 and 52.57, respectively, with a ratio of 5.60. It proves the occurrence of stronger coupling (see S14 for more details in Supplementary Information). The F–P resonances in peaks A, B, and C move from the VO_2_ layer to the MoS_2_ layer, where the VO_2_ layer mainly acts as a reflector (Figure S14a). Also, the mean value of the metasurface’s absorption in the visible range decreases from 0.75 to 0.74, opposite to the temperature dependence of the VO_2_/Au/Si structure’s absorption. The impedance analysis shows that the *Z* of low-*ε* state increases in the wavelength range of 646–756 nm and further away from 1, which is the main reason for the lower average absorption in the visible range (Figure 6b). In the low-*ε* state, a plasmon resonance peak P is formed in the NIR region, and the relationship between the plasmon resonance and the bulk MoS_2_ side length *L* is investigated, as shown in Figure 6c. The peak P red-shifts and increases bandwidth with increasing *L* of MoS_2_. It can be explained by the expansion of the magnetic field from the MoS_2_ layer to the VO_2_ layer (raised optical path) and by the diverging magnetic field (Figure S14b). As *L* increases, the peak P value increases and then decreases, reaching a maximum at *L* = 140 nm, which can be verified by the average optical field value trend (Figure S14c).

### 3.3 Modulation of structural color

**Figure 7:**
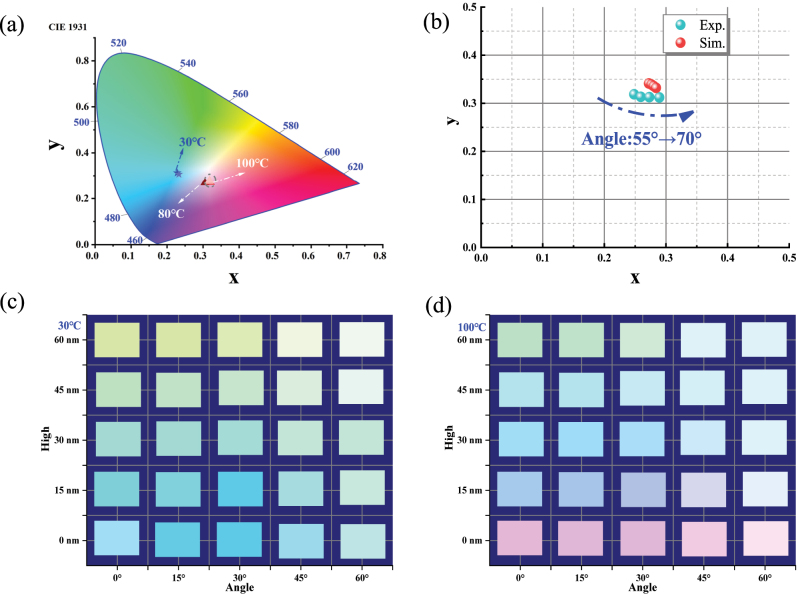
Structural color characteristics of the MoS_2_/VO_2_/Au/Si metasurface. (a) Data points in the CIE chromaticity diagram at different temperatures. The data in Figure S12a provide the raw spectral information. (b) Comparison of experimental data with FEM simulation data in *x*–*y* coordinates. The color palette of the reflective colors as a function of the thicknesses of MoS_2_ layers and oblique incidence angles at (c) 30 °C and (d) 100 °C.

The above results suggest that the temperature, oblique incidence angle, and morphology improve the metasurface system’s light–matter interactions and that structural color’s dynamic regulation potential is accordingly stimulated [34]. First, the effects of temperature and VO_2_ thickness on the structural color are investigated. Based on the temperature-dependent dielectric function, the reflectance spectra of the VO_2_/Au/Si structure in the visible range at different temperatures are obtained by dispersion relations [52]. The phase transition caused a blue shift in the spectrum with decreased absorption amplitude (Figure S15a). The data in the CIE chromaticity diagram intuitively represent the variations of color coordinates from (0.2281, 0.2128) to (0.3199, 0.2710) with increasing temperature from 30 °C to 100 °C (Figure 7a). Then, the color coordinates do not change with temperature. During this process, the brightness decreases from 0.9078 to 0.8784, with a change of 0.0294. The saturation decreases from 0.8724 to 0.3459, with a change of 0.5175. The color changes from aquamarine (Hue:8.7804°) to pink (Hue:128.185°). Figure 7b shows the color comparison between the FEM calculation and experiment, with an average error of 0.02415 in the coordinate positions, which is suitable for subsequent variable analyses. Next, we compared the difference in the effect of structural color on VO_2_ thickness due to phase transition by numerical calculation. More details of the calculations and analysis are shown in Supplementary Information S16. The results indicate that with increasing VO_2_ thickness at the normal incidence, the maximum variations of brightness and hue in the high-*ε* state are more prominent than that in the low-*ε* state, while the maximum variation of saturation is the opposite.

The sensitivities of the structural color of MoS_2_/VO_2_/Au/Si metasurfaces to the oblique incidence angles and heights of the MoS_2_ are investigated. Figure 7c shows that as the MoS_2_ height increases from *h* = 0 nm to *h* = 60 nm at an oblique incidence angle of 15°, the brightness increases from 0.32 to 0.4863 (*h* = 45 nm) and then decreases to 0.409 (*h* = 60 nm), with a maximum variation of 0.164. The hue falls from 183.5955° to 87.6893°, with a maximum variation of 96.206°. The saturation decreases from 1 to 0.1866, with a maximum variation of 0.8134. When the oblique incidence angle is 60°, the maximum variation values of brightness, hue, and saturation are 0.045, 49.5°, and 0.155, respectively. It indicates that the three attributes of color are all more sensitive to heights at low incidence angles. Fixed the height *h* = 15 nm, as the oblique incidence angle increases from 0° to 60°, the brightness first decreases from 0.4412 to 0.3863 (Angle = 30°) and then increases to 0.5686 (Angle = 60°), with a maximum variation of 0.1823. The hue increases from 176.5048° to 180° (Angle = 30°) and then decreases to 160.5°, with a maximum variation of 19.5°, corresponding to the turning trend of the variable angle spectra (Figure 5a–e). The saturation increases from 0.4578 to 0.4882 (Angle = 15°) and then decreases to 0.1818 with a maximum variation of 0.3064. When the height *h* = 60 nm, the maximum variation of brightness, hue, and saturation are 0.1647, 34.29°, and 0.1648, respectively. It indicates that the angular sensitivities of brightness and saturation are more significant at low heights, while the hue is the opposite. On the other hand, Figure 7 shows that the sensitivities of brightness and hue to height are more significant at low incidence angles in the low-*ε* state, while the trend for saturation is reversed. In addition, brightness and saturation are more sensitive to angle at low heights, while hue has the opposite direction. Furthermore, the sensitivities of structural color’ attributes to the temperature are consistent with the above findings (see S16 for more details in Supplementary Information). For example, the hue caused by the height of MoS_2_ in the low-*ε* state has a more pronounced change than the high-*ε* state, which can be visualized by comparing the color changes in the bottom two rows of Figure 7c and d, respectively. Table S2 in the Supplementary Information gives the RGB values under each structure with the corresponding detailed brightness, hue, and saturation values for the reader’s reference.

## 4 Conclusions

In summary, we present a MoS_2_/VO_2_/Au/Si metasurface with dynamic spectral tuning and reveal the modulation mechanism employing optical, magnetic field analysis, and impedance matching theory. The spectra of multilayer structures are first obtained by SE experiment. DFT results show that the onset of dielectric absorption peak K_2_ of VO_2_ is related to the O-2p state’s transition and has opposite responses to temperature in different phases. FEM simulations show that F–P resonance generated in VO_2_ and MoS_2_ dominates the metasurface’s absorption properties and is sensitive to the excitation of high temperature, oblique incidence angle, and morphology. Compared to the VO_2_ hierarchical structure, the metasurface can provide more than 2–3 times the absorption features, broaden the range of intense absorption, and increase the spectral absorption due to the high *ε″* properties and rich exciton features of MoS_2_. For example, an average absorption greater than 0.9 and near-perfect absorption of 0.9992 can be achieved in the Vis range. On the other hand, spectral perturbations in the Vis range affect the three attributes of the structural color. Most color performances exhibit higher sensitivity to external excitation in the high-*ε* state, at low oblique incidence angles, and at low heights of MoS_2_. Such design can also be applied to studying other TMDs materials coupled to VO_2_ or expanded into more complex material systems. This work offers fundamental ideas for enhancing the response to light and dynamic tuning of structural color, which can provide exciting applications for optical switching, tunable light detection, temperature sensing, etc.

## Supporting Information

Experimental dielectric functions of VO_2_, Au, and Si; details of the density functional theory (DFT) calculations and discussions; second-order derivative spectra of the absorption to wavelength; conditions for the formation of F–P resonance in VO_2_ and MoS_2_; polarization angles dependence and incidence angles dependence (TM and TE) of absorption; electromagnetic field distribution of metasurface at different oblique incidence angles and heights of MoS_2_; coupling of absorption peaks due to VO_2_ phase change; experimental spectra and structural colors in low-*ε* state; the RGB values under each structure with the corresponding detailed values of brightness, hue, and saturation.

## Supplementary Material

Supplementary Material Details
