# Supplementary material for: Dynamic tuning of optical absorbance and structural color of VO2-based metasurface
Source: Nanophotonics. 2023 Jun 12;12(15):3121–33. doi: 10.1515/nanoph-2023-0169 (PMC11501274; doi:10.1515/nanoph-2023-0169)
Supplement: Supplementary file 1 — Supplementary Material Details [file j_nanoph-2023-0169_suppl_001.docx]

**Supporting Information**

**Dynamic tuning of optical absorbance and structural color of VO_2_-based metasurface**

**TAO CHENG, YUKUAN MA, HUANHUAN ZHAO, TIANHAO FEI, LINHUA LIU^*^, AND JIA-YUE YANG^*^**

**Corresponding author: Jia-Yue Yang**: Optics & Thermal Radiation Research Center, Institute of Frontier and Interdisciplinary Science, Shandong University, Qingdao, 266237, P. R. China; and School of Energy and Power Engineering, Shandong University, Jinan, 250061, P. R. China, E-mail: jy_yang@sdu.edu.cn

**Linhua Liu**: Optics & Thermal Radiation Research Center, Institute of Frontier and Interdisciplinary Science, Shandong University, Qingdao, 266237, P. R. China; and School of Energy and Power Engineering, Shandong University, Jinan, 250061, P. R. China, E-mail: [liulinhua@sdu.edu.cn](mailto:liulinhua@sdu.edu.cn)

**Tao Cheng**: Optics & Thermal Radiation Research Center, Institute of Frontier and Interdisciplinary Science, Shandong University, Qingdao, 266237, P. R. China;

**Yukuan Ma**: College of Electronic Information, Sichuan University,Chengdu, 610000, P. R. China;

**Huanhuan Zhao**: School of Energy and Power Engineering, Shandong University, Jinan, 250061, P. R. China;

**Tianhao Fei**: School of Energy and Power Engineering, Shandong University, Jinan, 250061, P. R. China;

**S1 The scanning electron microscope (SEM) characteristics of VO_2_/Au/Si hierarchical structure**

The VO_2_/Au/Si hierarchical structure was characterized by surface with the scanning electron microscope (SEM) model SU8220. Here are SEM images of the multilayered film after and before VO_2_ deposition on Au, as shown in Figure S1a and Figure S1b, respectively. The VO_2_ film is polycrystalline, featured by many nanoparticles on top[1]. However, the Au film surface does not show a clear polycrystalline shape like VO_2_ film.

Figure S1 SEM image of the multilayered film (a) before and (b) after VO_2_ deposition on Au.

**S2 Crystal structure of VO_2_ in different phases**

**Figure S2** Crystal structure of VO_2_ in the (a) tetragonal rutile R and (b) monoclinic M_1_ phase.

**S3** **Experimental dielectric functions of VO_2_, Au, and Si**

As the phase transition temperature of VO_2_ is 68 ℃, the dielectric function at 80 ℃ shows a significant difference relative to 60 ℃ indicating the metallic phase properties (see Figures S2a-b). The phase transition reaches stability when the temperature is continued to 100 ℃, and the real part of the dielectric function starts to be less than 0 (-0.00298) at the wavelength *λ*=1235 nm. The experimental dielectric functions of Au and Si are shown in Figure S1 c-d. Due to the direct contact between Au and VO_2_ and the small thickness, the phase change of VO_2_ also causes a slight shift in the Au dielectric function. Figure S1e is the dielectric absorption of the different materials. The dielectric function of bulk MoS_2_ is available in the literature[2]. Three exciton peaks (Exciton1, 2, 3) are presented in the MoS_2_ dielectric function. The *εi* is the effective dielectric function of the nanostructured layer (MoS_2_) in air. It can be calculated from Maxwell-Garnett theory[3, 4]:

$\varepsilon i=\varepsilon_{nano}\frac{\varepsilon_{nano}(1+2f)+2\varepsilon_{nano}(1-f)}{\varepsilon_{env}(1-f)+\varepsilon_{nano}(2+f)}$ (S1)

where *f*=*V*_env_/*V*_0_ is the filling factor of the environmental media with *V*_0_= *V*_env_+ *V*_nano_. ε_env_ and ε_nano_ are the permittivity of the environment media and nanostructure material, respectively. When VO_2_ undergoes a high-temperature phase transition, a gradient refractive index distribution is formed in the wavelength range of 416 nm-697 nm.

**Figure S3** (a) Real part and (b) imaginary part of dielectric functions of VO_2_ at different temperatures obtained from ellipsometric experimental measurements. (c) Au’s Dielectric functions and (b) Si’s Dielectric functions at different temperatures obtained from ellipsometric experimental measurements. (e) Dielectric absorption of different materials.

**S4** **Details of the density functional theory (DFT) calculations and discussions**

We employ DFT approaches implemented in the Vienna ab initio Simulation Package (VASP)[5] to calculate bulk material properties for each phase of VO_2_. The exchange-correlation function was considered at the level of the GGA and specifically used the Perdew-Burke-Ernzerhof (PBE)[6] form. We correct the DFT bandgap and the electronic and vibrational properties of metal oxides (VO_2_) with the help of the DFT+U method[7, 8] (U is the Coulomb repulsion parameter). After referring to the literature values[9] and making appropriate adjustments, the adopted values of local Coulomb (U) and exchange interaction (J) in the DFT+U are 4.4 and 0.6 eV, respectively. The same U and J values are chosen for both phases for comparison purposes. We use experimentally obtained lattice parameters[10] for both systems and optimize internal atomic positions until the total energy of interatomic interactions is stable value. The *k*-mesh and the kinetic energy cutoff were set as 14×14×14 and 550 eV, respectively.

Figures S4a-d show the calculated electron energy bands and density of states (DOS). The V-3d state electrons and the O-2p state electrons show dominance in the interband and intraband transitions of the two phases. In the rutile (R) phase, the Fermi energy level overlaps the V-3d state electrons, thus giving a metallic state. V-3d electrons split into σ-bonded orbitals and π-bonded orbitals, the former hybridizing with the O atoms and the latter associated with crossing the Fermi level[11]. O-2p state electrons contribute mainly to the lower energy band (around -2 to -6 eV). In the M_1_ phase, the lattice distortion leads to the shifting of the electrons of the hybridized V-3d state, presenting a semiconductor indirect band gap of 0.5272 eV, which is consistent with our experiments (0.5296 eV). Specifically, V-V dimerization causes the splitting of the ɑ_1g_ band into bonding-antibonding subbands. The lowering of the bonding ɑ_1g_ band and the rise of the e_g_^π^ subband open the band gap at the Fermi level[11]. At this time, the O-2p electrons contribute mainly to the 0~-6eV region and the higher energy levels (>7eV). After adjusting the Fermi level to the 0 eV position, the V-3p state contributes mainly to the 0~6eV region as well as lower energy levels (<6eV).

**Figure S4** (a) R-phase electric energy band structure. (b) M_1_-phase electric energy band structure. (c) R-phase electric density of states (DOS). (d) The M_1_-phase electric density of states (DOS). (e) The dependence of the R phase’s band gap as a function of U_eff_-value and several types of representative literature values[11-13]. (f) The dependence of the M_1_ phase’s band gap as a function of U_eff_-value.

Then, we studied the effect of the values of U and J on the band gap of both phases. The two parameters U and J have been replaced by U_eff_ = U-J. Figure S4e shows the dependence of the band gap as a function of the U-value and comparison with literature values[11-13]. Stack et al. obtained an experimental value of 0.6 eV[12], Qazilbash et al. obtained a practical value of ≈ 0.5 eV[13], and Eaton et al. [11] obtained a simulated value of 0.45 eV. Increasing the U_eff_ can significantly increase the band gap of the M_1_ phase, which is attributed to the effective correction of the Coulomb and exchange interaction forces. The U_eff_ value can be assigned to a suitable empirical value by fitting the band structure to available experimental values[14]. However, as shown in Figure S4f, the correction of U_eff_ decreases the band gap of the R phase thus increasing its metallic character.

**S5** **Second-order derivative spectra of the absorption to wavelength (**$\frac{\boldsymbol{\partial}^{\boldsymbol{2}}\boldsymbol{A}}{\boldsymbol{\partial}\boldsymbol{\lambda}^{\boldsymbol{2}}}$**)**

**Figure S5** (a) The ($\frac{\partial^{2}A}{\partial\lambda^{2}}$) obtained from the FEM simulation of the “VO_2_/Au/Si” structure. (b) The ($\frac{\partial^{2}A}{\partial\lambda^{2}}$) obtained from the “VO_2_/Au/Si” structure experiment. (c) The ($\frac{\partial^{2}A}{\partial\lambda^{2}}$) obtained from the FEM simulation of the “VO_2_/Si” structure. (d) The ($\frac{\partial^{2}A}{\partial\lambda^{2}}$) obtained from the FEM simulation of the “Au” structure. (e) The ($\frac{\partial^{2}A}{\partial\lambda^{2}}$) obtained from the FEM simulation of the “VO_2_” structure.

**S6 The absorption of the hierarchical structure**

**Table S1** Comparison of absorption before and after phase transition of the hierarchical structure in the Vis and NIR range

|  | Average absorption | |
| --- | --- | --- |
| Wavelength | 0.38-1.3 µm | 0.38-0.76 µm |
| 30 ℃ | 0.52 | 0.64 |
| 100 ℃ | 0.69 | 0.66 |

The power absorption (*S*, W/m^3^) can be defined as[15]:

$w(x,y,z)=\frac{1}{2}\varepsilon_{0}\omega\varepsilon_{2}(x,y,z)\left|E(x,y,z)\right|^{2}$ (S2)

where *ε*_0_ is the vacuum dielectric constant, *ω* is the angular frequency.

**S7** **Variation of Fabry-Perot (F-P) resonance with thickness in VO_2_**

As the VO_2_ height decreases, the H distribution within the layer diminishes (Figure S6a, Figure S6c), which is understood as a decrease in the optical path. In particular, the magnetic field in the local area in Figure S6b has higher values, which explains the narrow bandwidth at thickness *t*=90 nm.

**Figure S6** (a) Magnetic field at *h*_VO2_=123 nm, *λ*=0.588 µm. (b) Magnetic field at *h*_VO2_=90 nm, *λ*=0.530 µm. (c) Magnetic field at *h*_VO2_=60 nm, *λ*=0.500 µm.

**S8** **Polarization angle dependence of absorption and electromagnetic field distribution at different oblique incidence angles of MoS_2_ nanostructure.**

Figures S7a-d show the variation of absorption with polarization angle for vertical and oblique incidence of the light source. The absorption has polarization dependence only at oblique incidence, and increases when the polarization direction is close to the TM direction. As shown in Figure S7e, the average values of the electric fields of Peak B in 0˚, 30˚, and 60˚ are 6.52E7, 6.29E7, and 5.54E7, respectively. It can be seen from the magnetic field distribution that the H at 60˚ has a more substantial divergence compared to that at 0˚, which confirms the result of a more divergent magnetic field at larger angles.

**Figure S7** (a) Wavelength-polarization angle contour map of absorption: *A*_total_ at (a) vertical and (b) 60° incidence angle, (c) *A*_TE_ at 60° incidence angle, (d) *A*_TM_ at 60° incidence angle. (e) The electric fields for peak B at different angles. (f) Magnetic fields of Peak X at different angles.

**S9** **Angle-dependent absorption contour plot under TE and TM directions**

The distribution of each absorption peak and dip are all marked in Figure S8. The TM direction's absorption has higher values than the TE direction and is the main source of contribution to dip X formation.

**Figure S8** Absorption contour plot under (a) TE and (b) TM direction.

**S10 Absorption and optical field of finite-thickness MoS_2_ and displacement of the absorption peaks with height**

**Figure S9** (a) Absorption of multi-layer MoS_2_ structures. (b) Optical field of multi-layers MoS_2_ structures (0, 1, and 4 layers) (c) ($\frac{\partial^{2}A}{\partial\lambda^{2}}$) obtained from the FEM simulation of “MoS_2_/VO_2_/Au/Si” structure at different heights.

**S11 Comparison of the magnetic field distribution of each peak at different MoS_2_ heights**

**Figure S10** Magnetic fields under vertical incidence when (a) *h*=50 nm and (b) *h*=70 nm.

**S12 Comparison of the magnetic and optical field distribution of each peak at different MoS_2_ lengths**

**Figure S11** Magnetic fields of peak N under vertical incidence at (a) *L*=152 nm, (b) *L*=180 nm, (c) *L*=202 nm. Optical fields of peak N under vertical incidence at (d) *L*=152 nm, (e) *L*=180 nm, (f) *L*=202 nm.

**S13 Comparison of the optical and electric fields between peak D′ and peak D**

The optical fields of both peak D′ and peak D are localized at the upper corner of MoS_2_. The magnetic fields of both are localized at the shoulder of MoS_2_ as well as at the central region of VO_2_. And there is a magnetic field deficiency in the central region of MoS_2_. These all prove the similarity of peak D′ with peak D.

**Figure S12** (a) The optical field of peak D´ (*λ*=536 nm) at *P*=250 nm, *L*=180 nm. (b) The optical field of peak D (*λ*=495 nm) under MoS_2_ nanostructure. (c) The magnetic field of peak D´ (*λ*=536 nm) at *P*=250 nm, *L*=180 nm. (d) The magnetic field of peak D (*λ*=495 nm) under MoS_2_ nanostructure.

**S14** **Coupling of absorption peaks due to VO_2_ phase change**

According to the literature[16], the quality factor is defined as:

$Q=\frac{\omega_{pl}}{\gamma_{pl}}$ (S3)

where *ω*_pl_ is the absorption peak frequency and γ_pl_ is the absorption peak broadening. In the uncoupled case, ω_pl_ =ω_A_ + ω_B_ - ω_B’_, and γ_pl_ =γ_A_ + γ_B_ - γ_B’_.

**Figure S13** Absorption of MoS_2_ nanostructure at 30 ℃ and 100 ℃.

**S15 Distribution of electric and magnetic fields in the low-state**

Compared to Figure S10a, Figure S14a shows that the magnetic fields (*λ*=595 nm, *λ*=645 nm, *λ*=705 nm) are more localized to the MoS_2_ layer, proving that F-P resonances are mainly dominated in MoS_2_ at this time. Figure S14b shows that the optical path is more significant at the edge length *L*=115 nm. Figure S11c shows that the mean value of the optical field at *L*=115 nm (7.62E7) is greater than that at lower (7.09E7) or higher (6.30E7) *L* values, indicating that there is a queue in the plasmon resonance absorption in the presence of increasing *L*.

**Figure 14** (a) Magnetic fields of different absorption peaks in low-ε state. (b) Magnetic fields of the plasmonic resonance peaks at different MoS_2_ volumes. (c) Optical field at peak (P) position (*λ*) for different MoS_2_ edge lengths (*L*).

**S16** **Spectra and structural colors in low-ε state**

Figures S15b-c show that the VO_2_ thickness affects the color attributes in the high-ε state. As the thickness increases from *h*=40 nm to 123 nm, the color shifts clockwise along the center point (0.33, 0.33). In particular, the brightness increases from 0.5667 to 0.7294 (*h*=80 nm) and then decreases to 0.3059 (*h*=123 nm) with a maximum variation of 0.4235. The hue reduces from 160.2976˚ to 15.5335˚ (*h*=80 nm), then increases to 326.3159˚ (*h*=100 nm), and finally decreases to 181.9233˚ with a maximum variation of 310.4824˚. The maximum change of saturation is 0.8472.

The color changes significantly by changing the VO_2_ height from when VO_2_ is in the low-ε state (Figure S15d-e). As the height increases from *h*=40 nm to 123 nm, the color converts clockwise from cyan to pick along the central point (0.33, 0.33). Specifically, the brightness increases from 0.302 at *h*=40 nm to 0.6549 (*h*=100 nm) and then decreases to 0.5294, with a maximum variation of 0.529. The hue reduces from 187.7291˚ to 44.5694˚ (*h*=80 nm), then increases to 353.0767˚ (*h*=100 nm), and finally decreases again to 296.6681˚, with a maximum variation of 308.5073˚. The maximum variation in saturation is 0.85.

**Figure S15** (a) Reflectance spectra of the VO_2_/Au/Si structure in the visible range at different temperatures. (b) Reflectance of high-ε state at different VO_2_ thicknesses. (c) Data points of high-ε state at different VO_2_ thicknesses in the CIE chromaticity diagram. (e) Reflectance of low-ε state at different VO_2_ thicknesses. (f) Data points of low-ε state at different VO_2_ thicknesses in the CIE chromaticity diagram.

The sensitivities of the color of the MoS_2_/VO_2_/Au/Si structure (low-ε) to the oblique incidence angles and heights were investigated. Figure 7d shows as the MoS_2_ height increases from *h*=0 nm to *h*=60 nm at an oblique incidence angle of 15˚, the brightness decreases from 0.5188 to 0.3569, with a maximum variation of 0.1619. The hue decreases from 290.2695˚ to 143.5738˚, with a maximum variation of 146.6959˚. The saturation first increases from 0.1486 to 0.2222 (*h*=30 nm) and then decreases to 0.1588, with a maximum variation of 0.0.0736. When the oblique incidence angle is 60˚, the maximum variation values of brightness, hue, and saturation are 0.0628, 140˚, and 0.0206, respectively. This proves that the height sensitivities of brightness and hue are more significant at low angles, while the saturation is the opposite. When the height *h*=15 nm, as the oblique incidence angle increases from 0˚ to 60˚, the brightness first decreases from 0.5235 to 0.4804 (Angle=30˚) and then increases to 0.55765 (Angle=60˚), with a maximum variation of 0.1965. The hue increases from 212.94˚ to 223.6351˚ (Angle=45˚) and then decreases to 205.7158˚, with a maximum variation of 17.9193˚. The saturation decreases from 0.2099 to 0.0648, with a maximum variation of 0.1451. When the height h=60 nm, the maximum brightness, hue, and saturation variation are 0.1686, 37.5042˚, and 0.1027, respectively. It proves that the angular sensitivities of brightness and saturation are more significant at low heights, while the hue is the opposite.

**S17** **The RGB values under each structure with the corresponding detailed values of brightness, hue, and saturation**

**Table S2** The RGB and three attributes of structural color

| Structure | Parameters | R | G | B | Brightness | hue | Saturation |
| --- | --- | --- | --- | --- | --- | --- | --- |
| 30℃-15° | *H*=0nm | 0 | 144 | 154 | 0.302 | 183.8955 | 1 |
|  | *H*=15nm | 54 | 157 | 155 | 0.4137 | 178.834 | 0.4882 |
|  | *H*=30nm | 92 | 155 | 141 | 0.4843 | 166.6672 | 0.2551 |
|  | *H*=45nm | 106 | 142 | 117 | 0.4863 | 138.3311 | 0.1452 |
|  | *H*=60nm | 106 | 124 | 85 | 0.4098 | 87.6893 | 0.1866 |
| 30-60° | *H*=0nm | 116 | 157 | 150 | 0.5353 | 169.7568 | 0.173 |
|  | *H*=15nm | 125 | 165 | 152 | 0.5686 | 160.4998 | 0.1818 |
|  | *H*=30nm | 135 | 161 | 149 | 0.5804 | 152.3068 | 0.1215 |
|  | *H*=45nm | 138 | 152 | 144 | 0.5686 | 145.7158 | 0.0636 |
|  | *H*=60nm | 137 | 143 | 137 | 0.549 | 120 | 0.0261 |
| 30℃-*h*=15nm | 0° | 61 | 164 | 158 | 0.4412 | 176.5048 | 0.4578 |
|  | 15° | 54 | 157 | 155 | 0.4137 | 178.834 | 0.4882 |
|  | 30° | 54 | 143 | 143 | 0.3863 | 180 | 0.4518 |
|  | 45° | 88 | 142 | 135 | 0.451 | 172.2223 | 0.2348 |
|  | 60° | 125 | 165 | 152 | 0.5686 | 160.4998 | 0.1818 |
| 30℃-*h*=60nm | 0° | 113 | 131 | 89 | 0.4314 | 85.7143 | 0.1909 |
|  | 15° | 106 | 124 | 85 | 0.4098 | 87.6893 | 0.1866 |
|  | 30° | 98 | 113 | 83 | 0.3843 | 90.0025 | 0.1531 |
|  | 45° | 113 | 119 | 107 | 0.4431 | 90 | 0.0531 |
|  | 60° | 137 | 143 | 137 | 0.549 | 120 | 0.0261 |
| 100℃-15˚ | *H*=0nm | 143 | 112 | 149 | 0.5118 | 290.2695 | 0.1486 |
|  | *H*=15nm | 105 | 125 | 155 | 0.5098 | 215.9976 | 0.2 |
|  | *H*=30nm | 91 | 128 | 143 | 0.4588 | 197.3068 | 0.2222 |
|  | *H*=45nm | 83 | 120 | 120 | 0.398 | 180 | 0.1823 |
|  | *H*=60nm | 77 | 105 | 88 | 0.3569 | 143.5738 | 0.1538 |
| 100℃-60˚ | *H*=0nm | 152 | 140 | 148 | 0.5726 | 320.0042 | 0.055 |
|  | *H*=15nm | 140 | 148 | 154 | 0.5765 | 205.7158 | 0.0648 |
|  | *H*=30nm | 134 | 148 | 151 | 0.5588 | 190.5925 | 0.0756 |
|  | *H*=45nm | 127 | 143 | 145 | 0.5333 | 186.6723 | 0.0756 |
|  | *H*=60nm | 122 | 138 | 138 | 0.5098 | 180 | 0.064 |
| 100℃-*h*=15nm | 0° | 108 | 131 | 159 | 0.5235 | 212.94 | 0.2099 |
|  | 15° | 105 | 125 | 155 | 0.5098 | 215.9976 | 0.2 |
|  | 30° | 102 | 114 | 143 | 0.4804 | 222.4381 | 0.1673 |
|  | 45° | 114 | 120 | 136 | 0.4902 | 223.6351 | 0.088 |
|  | 60° | 140 | 148 | 154 | 0.5765 | 205.7158 | 0.0648 |
| 100℃-*h*=60nm | 0° | 80 | 112 | 92 | 0.3765 | 142.4958 | 0.1667 |
|  | 15° | 77 | 105 | 88 | 0.3569 | 143.5738 | 0.1538 |
|  | 30° | 78 | 96 | 85 | 0.3412 | 143.3319 | 0.1035 |
|  | 45° | 96 | 109 | 107 | 0.402 | 170.7729 | 0.0634 |
|  | 60° | 122 | 138 | 138 | 0.5098 | 180 | 0.064 |
| 30℃-0˚ | *H*=40nm | 111 | 178 | 156 | 0.5667 | 160.2976 | 0.3032 |
| (Without MoS_2_) | *H*=60nm | 111 | 178 | 156 | 0.5667 | 160.2976 | 0.3032 |
|  | *H*=80nm | 222 | 169 | 150 | 0.7294 | 15.8335 | 0.5217 |
|  | *H*=100nm | 181 | 124 | 156 | 0.598 | 326.3159 | 0.278 |
|  | *H*=110nm | 123 | 127 | 158 | 0.551 | 233.1415 | 0.1528 |
|  | *H*=123nm | 0 | 151 | 156 | 0.3059 | 181.9223 | 1 |
| 100℃-0˚ | *H*=40nm | 0 | 134 | 154 | 0.302 | 187.7921 | 1 |
| (Without MoS_2_) | *H*=60nm | 128 | 175 | 155 | 0.5941 | 154.468 | 0.227 |
|  | *H*=80nm | 182 | 173 | 147 | 0.6451 | 44.5694 | 0.1934 |
|  | *H*=100nm | 193 | 141 | 147 | 0.6549 | 353.0767 | 0.2955 |
|  | *H*=110nm | 183 | 124 | 150 | 0.602 | 333.5578 | 0.2906 |
|  | *H*=123nm | 151 | 117 | 153 | 0.5294 | 296.6681 | 0.15 |
| VO_2_-30℃ | 0˚-*h*_VO2_=123nm | 252 | 217 | 211 | 0.9078 | 8.7804 | 0.8724 |
| VO_2_-100℃ | 0˚-*h*_VO2_=123nm | 213 | 235 | 216 | 0.8784 | 128.185 | 0.3549 |

**Reference**

[1] F. Shu, F. Yu, R. Peng, Y. Zhu, B. Xiong, R. Fan, Z. Wang, Y. Liu, and M. Wang, “Dynamic Plasmonic Color Generation Based on Phase Transition of Vanadium Dioxide,” *Adv. Opt. Mater.,* vol. 6, pp.1700939, 2018.

[2] A. S. Sarkar, A. Mushtaq, D. Kushavah, and S. K. Pal, “Liquid exfoliation of electronic grade ultrathin tin(II) sulfide (SnS) with intriguing optical response,” *NPJ 2D Mater. Appl.,* vol. 4, no. 1, pp. 1, 2020.

[3] M. Wang, C. Hu, M. Pu, C. Huang, Z. Zhao, Q. Feng, and X. Luo, “Truncated spherical voids for nearly omnidirectional optical absorption,” *Opt Express,* vol. 19, no. 21, pp. 20642-20649, 2011.

[4] Y. Huang, L. Liu, M. Pu, X. Li, X. Ma, and X. Luo, “A refractory metamaterial absorber for ultra-broadband, omnidirectional and polarization-independent absorption in the UV-NIR spectrum,” *Nanoscale,* vol. 10, no. 17, pp. 8298-8303, 2018.

[5] G. Kresse, and J. Furthmüller, “Efficient iterative schemes for ab initio total-energy calculations using a plane-wave basis set,” *Phys. Rev. B,* vol. 54, no. 16, pp. 11169-11186, 1996.

[6] J. P. Perdew, K. Burke, and M. Ernzerhof, “Generalized Gradient Approximation Made Simple,” *Phys. Rev. Lett,* vol. 77, no. 18, pp. 3865-3868, 1996.

[7] L. A. Agapito, S. Curtarolo, and M. Buongiorno Nardelli, “Reformulation of DFT+U as a Pseudohybrid Hubbard Density Functional for Accelerated Materials Discovery,” *Phys. Rev. X,* vol. 5, no. 1, pp. 011006, 2015.

[8] P. Gopal, M. Fornari, S. Curtarolo, L. A. Agapito, L. S. I. Liyanage, and M. B. Nardelli, “Improved predictions of the physical properties of Zn- and Cd-based wide band-gap semiconductors: A validation of the ACBN0 functional,” *Phys. Rev. B,* vol. 91, no. 24, pp. 245202, 2015.

[9] A. Liebsch, H. Ishida, and G. Bihlmayer, “Coulomb correlations and orbital polarization in the metal-insulator transition of VO2,” *Phys. Rev. B,* vol. 71, no. 8, pp. 085109, 2005.

[10] D. Kucharczyk, and T. Niklewski, “Accurate X-ray determination of the lattice parameters and the thermal expansion coefficients of VO2 near the transition temperature,” *J Appl Crystallogr,* vol. 12, no. 4, pp. 370-373, 1979.

[11] M. Eaton, A. Catellani, and A. Calzolari, “VO(2) as a natural optical metamaterial,” *Opt Express,* vol. 26, no. 5, pp. 5342-5357, 2018.

[12] J. D. Stack, and R. J. Wensley, “Large loops of magnetic current and confinement in four dimensional U(1) lattice gauge theory,” *Phys. Rev. Lett,* vol. 72, no. 1, pp. 21-24, 1994.

[13] M. M. Qazilbash, M. Brehm, B.-G. Chae, P.-C. Ho, G. O. Andreev, B.-J. Kim, S. J. Yun, A. V. Balatsky, M. B. Maple, F. Keilmann, H.-T. Kim, and D. N. Basov, “Mott Transition in VO2 Revealed by Infrared Spectroscopy and Nano-Imaging,” *Science,* vol. 318, no. 5857, pp. 1750-1753, 2007.

[14] B. Himmetoglu, A. Floris, S. de Gironcoli, and M. Cococcioni, “Hubbard-corrected DFT energy functionals: The LDA+U description of correlated systems,” *Int J Quantum Chem,* vol. 114, no. 1, pp. 14-49, 2014.

[15] J. M. Zhao, and Z. M. Zhang, “Electromagnetic energy storage and power dissipation in nanostructures,” *J Quant Spectrosc Radiat Transf,* vol. 151, pp. 49-57, 2015.

[16] G. Zengin, M. Wersäll, S. Nilsson, T. J. Antosiewicz, M. Käll, and T. Shegai, “Realizing Strong Light-Matter Interactions between Single-Nanoparticle Plasmons and Molecular Excitons at Ambient Conditions,” *Phys. Rev. Lett,* vol. 114, no. 15, pp. 157401, 2015.
